# Exposure to e-cigarette and heated tobacco product advertisements via digital, traditional media, and points-of-sale: An examination of associations with use intentions and perceived risk among adults in Armenia and Georgia

**DOI:** 10.18332/tpc/191992

**Published:** 2024-10-25

**Authors:** Zhanna Sargsyan, Ana Dekanosidze, Varduhi Hayrumyan, Arevik Torosyan, Yuxian Cui, Lilit Grigoryan, Nour Alayan, Varduhi Petrosyan, Alexander Bazarchyan, Lela Sturua, Regine Haardörfer, Michelle C. Kegler, Carla J. Berg

**Affiliations:** 1 Turpanjian College of Health Sciences, American University of Armenia, Yerevan, Armenia; 2 Georgia National Center for Disease Control and Public Health, Tbilisi, Georgia; 3 Tbilisi State Medical University, Tbilisi, Georgia; 4 National Institute of Health named after academician S. Avdalbekyan, Ministry of Health of the Republic of Armenia, Yerevan, Armenia; 5 Department of Prevention and Community Health, Milken Institute School of Public Health, The George Washington University, Washington, United States; 6 Department of Behavioral, Social, and Health Education Sciences, Rollins School of Public Health, Emory University, Atlanta, United States; 7 George Washington Cancer Center, The George Washington University, Washington, United States

**Keywords:** e-cigarettes, tobacco industry, heated tobacco products, risk perceptions, tobacco marketing

## Abstract

**INTRODUCTION:**

E-cigarette and heated tobacco product (HTP) marketing often leverages digital media and points-of-sale (POS) and advertises risk reduction, including in Armenia and Georgia where male cigarette use rates are high.

**METHODS:**

Using 2022 survey data from Armenian and Georgian adults (n=1468, mean age=42.92 years, 51.4% female; and past-month use of e-cigarettes 3.2%, HTPs 2.7%, and cigarettes 31.6%), multivariable linear regression examined 4 outcomes – e-cigarette and HTP use intentions and perceived risk (1=not at all, to 7=extremely) – in relation to past-month e-cigarette or HTP advertisement exposure via digital media, traditional media, and POS, controlling for covariates (country, age, gender, education level, relationship status, children, past-month cigarette and e-cigarette/HTP use).

**RESULTS:**

E-cigarette and HTP use intentions were low (mean score=1.47, SD=1.39 each), while perceived risk was high (mean score=5.83, SD=1.6, and mean score=5.87, SD=1.56, respectively). Past-month exposure to e-cigarette and HTP advertisements, respectively, were 12.9% and 11.2% via digital media, 6.1% and 4.8% traditional media, and 22.5% and 21.1% POS. For e-cigarettes, ad exposure via digital media was associated with greater use intentions (β=0.24; 95% CI: 0.03–0.44), ad exposure via traditional media (β= -0.32; 95% CI: -0.55 – -0.09) and POS (β= -0.30; 95% CI: -0.60 – -0.004) was associated with lower risk perceptions. For HTPs, ad exposure via digital media (β=0.35; 95% CI: 0.14–0.56) and POS (β=0.21; 95% CI: 0.04–3.63) was associated with greater use intentions, and ad exposure at POS was associated with lower risk perceptions (β= -0.23; 95% CI: -0.42 – -0.03).

**CONCLUSIONS:**

Tobacco control efforts should monitor and regulate e-cigarette and HTP marketing, particularly via digital media which may effectively promote use, and via POS which may target and influence risk perceptions.

## INTRODUCTION

In recent years, the global tobacco market has significantly diversified, largely due to the emergence of e-cigarettes and heated tobacco products (HTPs)^1^. These products have been aggressively marketed as harm reduction products and using various channels, including traditional media, digital media, and points-of-sale (POS)^2-4^. Exposure to e-cigarette and HTP marketing may impact how people perceive, use, and intend to use e-cigarettes and HTPs^5-8^. For example, a 2014 study of US adolescents documented that e-cigarette TV advertisement exposure increased e-cigarette use and use intentions^5^. Another study of US college students found that positive appraisal of e-cigarette advertising videos was associated with e-cigarette use intentions^6^. A 2020 study of South Korean adults showed that e-cigarette and HTP advertisement exposure was associated with perceiving them less harmful compared with cigarettes^7^.

E-cigarette and HTP marketing has diversified, shifting from traditional media to POS and digital platforms, adapting to changing consumer preferences, technological advancements, and tobacco control regulations. Certain marketing channels may be more effective in shifting consumer perceptions and promoting use. POS advertisements strategically influence consumer decisions within the retail setting and often emphasize reduced harm claims^9^. Digital media, including websites, social media, and mobile apps, allow for precise targeting and interactive engagement^10^, and one study found that e-cigarette-related posts made by sponsored users on social media are perceived as more trustworthy and authentic than posts made by brands’ official accounts or traditional advertising^11^. Notably, analysis of data from the International Tobacco Control (ITC) Project in Japan^12^ and South Korea^7^ found that being exposed to HTP advertising via traditional media (e.g. television, radio, posters/billboard, newspapers), digital media (e.g. social media), and/or POS was associated with perceiving HTPs as less harmful than cigarettes. Additionally, there is a wealth of research showing associations between digital e-cigarette advertising exposure and reduced e-cigarette related harm perceptions^13^ and e-cigarette use over time among young and older adults^6,14-17^. Unfortunately, effectively regulating digital marketing of e-cigarettes and HTPs represents a particular challenge^4,18,19^, due to global accessibility, gaps in regulatory frameworks, difficulties in enforcement, and innovative approaches used by the industry^20^.

The marketing of novel tobacco products, such as e-cigarettes and HTPs, in low- and middle-income countries (LMICs) is a growing global health concern. Tobacco companies are increasingly targeting these regions, as many of these countries represent untapped markets, consumers enticed by harm reduction claims, and weaker regulatory frameworks^21^. Armenia and Georgia, two middle-income countries in the South Caucasus region, serve as intriguing case studies for examining the impact of e-cigarette and HTP marketing. Despite cultural differences and distinct histories, Armenia and Georgia share a common characteristic – high rates of cigarette use among men (56.1% and 49.5%, respectively), but much lower rates (2.6% and 8.5%) among women^22,23^. Additionally, prevalence of traditional cigarette smoking in Armenia and Georgia has presented a unique context for the introduction and marketing of alternative products. Armenia’s revenue in the e-cigarettes market is projected to reach US$7.0 million and in Georgia US$10.1 million in 2023, with an annual growth rate of 3.15% and 2.85%, respectively^24^.

Armenia and Georgia have comprehensive bans on tobacco advertising and promotion (in Armenia since 2020 and Georgia since 2018). In Armenia, regulations implemented in January 2020 prohibit all forms of advertising, promotion, sales, and sponsorship of tobacco products, and POS display bans on tobacco products and their accessories, substitutes, and imitations, including packaging, posters, or trademarks, have been in place since January 2022^25^. In Georgia, policies implemented and strengthened since 2018 have been equally comprehensive, although products that do not contain nicotine, like some e-cigarettes, are not captured in the legal definition of tobacco products. Despite comprehensive laws, compliance is limited, given the common enforcement challenges faced across LMICs (e.g. less resources for enforcement, greater tobacco industry interference)^26^.

This study fills critical gaps in understanding the impact of e-cigarette and HTP marketing on use intentions and risk perceptions among adults in Armenia and Georgia, where male cigarette usage rates are notably high. The existing literature is mostly limited to adolescents and young adults; this study will add the perspective of adult population to the literature. We hypothesized the exposure to e-cigarette and HTP advertising will increase use intentions and decrease perceived risk. The study findings inform monitoring and regulation of marketing strategies for these products, particularly focusing on digital media and POS, to strengthen tobacco control efforts and mitigate the potential impact on public health.

## METHODS

### Study overview

The parent study (launched in Fall 2018) used a matched-pairs community randomized controlled trial involving 28 communities in Armenia and Georgia to examine the effectiveness of local coalitions in promoting smoke-free air and reducing secondhand smoke exposure (SHSe)^27^.

### Data collection

In each of the 28 communities (intervention and control), we conducted population-level surveys at baseline in October–November 2018 and at follow-up in May–June 2022. Current analyses focused on the 2022 survey data. Sampling strategies were different across countries because of availability of household data in Armenia (but not Georgia) and the utility of ‘clusters’ (i.e. geographically defined areas of 150 households) in Georgia (but not Armenia). In both countries, we obtained census data for households within the municipality limits, then used the Kish method to identify target participants (i.e. ages 18–64 years) in each household to reach target recruitment (n=50 per city)^27^.

In Armenia, addresses in each city were randomly ordered (using a random number generator); assessments began at the beginning of the list and continued until recruitment targets were reached. In 2022, 1140 households were visited; of the 890 (78.1%) eligible, 763 (85.7%) participated. In Georgia, 5 clusters per city were identified, then 15 households per cluster were selected using a random walking method. In 2022, 916 households were visited; of the 839 (91.6%) eligible, 705 (84.0%) participated.

### Measures

The survey was developed in English and translated/back-translated for the Armenian and Georgian languages.

*Primary outcomes: e-cigarette and HTP use intentions and risk perceptions*

E-cigarette and HTP use intentions, respectively, were assessed by asking: ‘How likely are you to try or continue to use electronic cigarettes or e-cigarettes, and heated tobacco products such as IQOS, in the next year?’ (1=not at all, to 7=extremely). E-cigarette and HTP risk perceptions were assessed by asking: ‘How harmful to your health and addictive do you think the electronic cigarettes or e-cigarettes and heated tobacco products such as IQOS, are?’ (1=not at all, to 7=extremely). Scores from the 2 items assessing e-cigarettes and the 2 items assessing HTPs, respectively, were averaged to create risk perception index scores (e-cigarette items, r=0.61; HTP items, r=0.62).

*Correlates of interest: e-cigarette and HTP media exposure*

Participants were asked: ‘This item asks about advertisements, meaning ads made and paid for by companies who make the products. In the last 30 days, have you noticed advertisements for e-cigarettes or vaping products, and for heated tobacco products like IQOS, in any of the following places?’ (Check all that apply): On websites (for example, pop-up ads); On social media sites, like Facebook, Instagram or Twitter; Inside stores that sell cigarettes and other tobacco products; Outside stores that sell cigarettes and other tobacco products (including on signs in windows, visible from the outside); Seeing specialty stores that sell vaping products and heated tobacco products; On television; On the radio; On posters, billboards, etc.; In newspapers or magazines; Direct mail; Email; Other (specify); None of the above. Responses from these questions were used to create 6 variables indicating any ad exposure for e-cigarettes and HTPs, respectively, via digital media (websites, social media), traditional media (TV, radio, posters/billboards, newspapers/magazines), or at points-of-sale (POS; inside or outside stores that sell tobacco products or specialty stores). There were few responses to direct mail, email, and other, so these were not categorized (see Table 1 footnote).

**Table 1:** Descriptive statistics regarding exposure to e-cigarettes and HTPs, cross-sectional survey of adults in Armenia and Georgia, 2022 (N=1468)

	*E-cigarettes*	*HTPs*
	*n (%)*	*n (%)*
mFirst exposure to product*^#^	1096 (74.7)	973 (66.3)
Saw products or advertisements (i.e. paid ads) in stores	177 (12.1)	168 (11.4)
Saw ads on TV, in magazines/newspapers, or on Internet/social media	83 (5.7)	70 (4.8)
Saw posts on social media (not ads)	77 (5.2)	44 (3.0)
Heard about them from friends, family, or co-workers	630 (42.9)	594 (40.5)
Saw them used on TV, movies, etc.	115 (7.8)	91 (6.2)
Other	14 (1.0)	6 (0.4)
Had not heard of product*	348 (23.7)	467 (31.8)
Past-month product* ad exposure^†^		
**Digital media**		
On websites (for example, pop-up ads)	64 (4.4)	57 (3.9)
On social media sites, like Facebook, Instagram or Twitter	155 (10.6)	139 (9.5)
Any digital media exposure	189 (12.8)	165 (11.2)
**Traditional media**		
On television	72 (4.9)	54 (3.7)
On the radio	5 (0.3)	2 (0.1)
On posters, billboards, etc.	15 (1.0)	14 (1.0)
In newspapers or magazines	7 (0.5)	5 (0.3)
Any traditional media exposure	90 (6.1)	71 (4.9)
**Points-of-sale**		
Inside stores that sell cigarettes and other tobacco products	265 (18.1)	254 (17.3)
Outside stores that sell cigarettes and other tobacco products	92 (6.3)	87 (5.9)
Seeing specialty stores that sell heated tobacco products	56 (3.8)	63 (4.3)
Any point-of-sale exposure	330 (22.5)	310 (21.1)
None	849 (57.8)	883 (60.1)
Product* use factors		
Lifetime use	158 (10.8)	109 (7.4)
Past-month use	47 (3.2)	39 (2.7)
Next-year use intentions, mean (SD)	1.47 (1.39)	1.47 (1.39)
**Product risk perceptions,** mean (SD)^§^	**5.83 (1.60)**	**5.87 (1.56)**
Addictiveness	5.93 (1.74)	5.96 (1.70)
Harm to health	5.73 (1.82)	5.78 (1.77)

Reported percentages include missing values.

* Product referencing e-cigarettes or HTPs, respectively.

# Other: Refuse: 1 for e-cigarettes, 0 for HTPs. Don’t know: 21 for e-cigarettes, 26 for HTPs. Missing: 0 for e-cigarettes, 2 for HTPs.

† Direct mail and email: 1 each for e-cigarettes, none for HTPs. Other: 9 for e-cigarettes, 7 for HTPs. Refuse: 11 for e-cigarettes, 14 for HTPs. Don’t know: 94 for e-cigarettes, 106 for HTPs; regrouped as ‘none’ for regression analyses.

§ Correlations for risk perception items: e-cigarette addictiveness and harm = 0.61; HTP addictiveness and harm = 0.62.

To characterize source of first product exposure, participants were also asked: ‘How did you first learn about e-cigarettes and heated tobacco products such as IQOS?’. Response options were: Saw products or advertisements (i.e. paid ads) in stores; Saw advertisements on TV, in magazines/newspapers, or on the Internet or social media; Saw posts on social media (not ads); Heard about them from friends, family, or co-workers; Saw them used on TV, movies, etc.; Other (please specify); and I have not heard of these.

*Covariates: sociodemographics and tobacco use*

Current analyses included the following covariates: country, age, sex, education level, employment status, relationship status, children in the home, and past 30-day cigarette, e-cigarette, and HTP use.

### Data analysis

Descriptive analyses were conducted to characterize participants and their responses to items regarding e-cigarettes and HTPs, respectively. Then, bivariate analyses (t-tests and one-way ANOVAs for categorical variables, Pearson’s r for continuous variables) were used to examine associations between past-month media exposure and e-cigarette and HTP use intentions and risk perceptions. Next, we conducted 4 multilevel linear regression models (accounting for clustering within communities) to identify correlates of e-cigarette and HTP use intentions and risk perceptions. Models for e-cigarette use intentions and risk perceptions included e-cigarette ad exposure variables and past-month e-cigarette use; models for HTP use intentions and risk perceptions included HTP ad variables and past-month HTP use; all models included sociodemographics and current cigarette use status. Employment was not included in the models, as education level and employment were highly correlated, and education showed greater associations with outcomes. Analyses were conducted in SPSS v.27, and alpha was set at 0.05.

## RESULTS

### Participant characteristics

In this sample (n=1468), the majority were from Armenia (52.0%), female (51.4%), high school educated or higher (73.1%), employed (61.3%), and married/cohabitating (66.5%); and less than half had children aged <18 years in the home (49.3%). Lifetime cigarette, e-cigarette, and HTP use were 39.5%, 10.8% and 7.4%, respectively. Past-month cigarette, e-cigarette, and HTP use were 31.6%, 3.2%, and 2.7%, respectively. Overall score for e-cigarette and HTP use intentions was 1.47 (SD=1.39), and perceived risk was 5.83 (SD=1.60) for e-cigarettes and 5.87 (SD=1.56) for HTPs. Sample characteristics are presented in Supplementary file Table 1.

### E-cigarette and HTP exposure

Table 1 represents descriptive statistics regarding exposure to e-cigarettes and HTPs. Overall, 74.7% and 66.3% have ever heard of e-cigarettes and HTPS, respectively. The most common source of first exposure to product included friends, family, or co-workers (42.9%, 40.5%, respectively), followed by advertisements in stores (12.1%, 11.4%), seeing them used on TV, movies (7.8%, 6.2%), seeing ads on TV, in magazines/newspapers, or on Internet/social media (5.7%, 4.8%), and seeing posts (not ads) on social media (5.2%, 3.0%) for e-cigarettes and HTPs, respectively. Past-month exposure to e-cigarette and HTP advertisements, respectively, were 12.8% and 11.2% via digital media, 6.1% an 4.9% via traditional media, and 22.5% and 21.1% at POS.

### Advertising exposure in relation to e-cigarette and HTP use intentions

In bivariate analyses, advertising exposure via digital media and POS was associated with significantly higher use intentions for both e-cigarettes (digital media: 1.87 vs 1.41, p<0.001; POS: 1.88 vs 1.35, p<0.001) and HTPs (digital media: 2.07 vs 1.39, p<0.001; POS: 1.99 vs 1.32, p<0.001). Other factors associated with greater use intentions for both e-cigarettes and HTPs included lifetime and past-month use of the respective product, past-month cigarette use, lower risk perceptions of the respective product, and being younger, male, less educated, unmarried/not cohabitating, and without children in the home (all p<0.05) (Table 2).

**Table 2:** Unadjusted bivariate analyses assessing preliminary associations between correlates of interest and e-cigarette and HTP use intentions and risk perceptions, cross-sectional survey of adults in Armenia and Georgia, 2022 (N=1468)

	*Use Intentions*				*Risk Perceptions*			
	*E-cigarettes*		*HTPs*		*E-cigarettes*		*HTPs*	
	*Mean (SD)*	*p*	*Mean (SD)*	*p*	*Mean (SD)*	*p*	*Mean (SD)*	*p*
First exposure to product*		**<0.001**		**<0.001**		**<0.001**		**<0.001**
Saw products or advertisements (i.e. paid ads) in stores	1.72 (1.71)		1.83 (1.98)		5.81 (1.72)		5.77 (1.69)	
Saw ads on TV, in magazines/newspapers, or on Internet/social media	1.33 (1.13)		1.49 (1.38)		5.55 (1.55)		5.58 (1.36)	
Saw posts on social media (not ads)	1.51 (1.48)		2.34 (2.20)		5.65 (1.62)		5.40 (1.89)	
Heard about them from friends, family, or co-workers	1.67 (1.61)		1.64 (1.56)		5.51 (1.68)		5.53 (1.66)	
Saw them used on TV, movies, etc.	1.28 (1.04)		1.31 (1.02)		6.00 (1.52)		5.97 (1.55)	
Had not heard of product*	1.07 (0.59)		1.07 (0.57)		6.49 (1.16)		6.42 (1.18)	
Past-month product* ad exposure								
**Digital media** – Yes	1.87 (1.68)	**<0.001**	2.07 (1.85)	**<0.001**	5.64 (1.57)	0.090	5.72 (1.60)	0.199
No	1.41 (1.33)		1.39 (1.30)		5.85 (1.60)		5.89 (1.56)	
**Traditional media** – Yes	1.68 (1.61)	0.140	1.61 (1.50)	0.386	5.57 (1.88)	0.111	5.70 (1.77)	0.365
No	1.45 (1.37)		1.46 (1.39)		5.84 (1.58)		5.88 (1.55)	
**Points-of-sale** – Yes	1.88 (1.90)	**<0.001**	1.99 (2.03)	**<0.001**	5.50 (1.87)	**<0.001**	5.38 (1.86)	**<0.001**
No	1.35 (1.17)		1.32 (1.12)		5.92 (1.50)		6.00 (1.45)	
Product* use factors								
**Lifetime use** – Yes	3.01 (2.21)	**<0.001**	3.50 (2.39)	**<0.001**	4.89 (1.83)	**<0.001**	4.85 (1.79)	**<0.001**
No	1.28 (1.12)		1.30 (1.13)		5.94 (1.53)		5.95 (1.52)	
**Past-month use** – Yes	4.19 (2.30)	**<0.001**	5.13 (2.13)	**<0.001**	4.20 (1.87)	**<0.001**	4.31 (1.89)	**<0.001**
No	1.38 (1.25)		1.37 (1.22)		5.88 (1.56)		5.91 (1.53)	
**Next-year use intentions,** r	-	-	-	-	-0.26	**<0.001**	-0.29	**<0.001**
Product* risk perceptions, r	-0.26	**<0.001**	-0.29	**<0.001**	-	**-**	-	**-**
**Past-month cigarette use** – Yes	2.08 (1.90)	**<0.001**	2.04 (1.87)	**<0.001**	4.95 (1.85)	**<0.001**	5.02 (1.84)	**<0.001**
No	1.18 (0.94)		1.20 (1.00)		6.23 (1.28)		6.26 (1.24)	
**Sociodemographics**								
**Country** – Armenia	1.47 (1.44)	0.987	1.46 (1.43)	0.867	5.86 (1.63)	0.410	5.92 (1.58)	0.156
Georgia	1.47 (1.33)		1.47 (1.35)		5.79 (1.57)		5.81 (1.54)	
**Age,** r	-0.14	**<0.001**	-0.13	**<0.001**	0.06	**0.015**	0.05	0.064
**Gender –** Male	1.77 (1.67)	**<0.001**	1.77 (1.68)	**<0.001**	5.30 (1.77)	**<0.001**	5.37 (1.75)	**<0.001**
Female	1.19 (0.98)		1.18 (0.97)		6.32 (1.23)		6.34 (1.18)	
**Education level** – ≤High school	1.58 (1.55)	0.072	1.58 (1.57)	0.055	5.58 (1.76)	**<0.001**	5.62 (1.72)	**<0.001**
>High school	1.43 (1.32)		1.42 (1.32)		5.92 (1.53)		5.96 (1.49)	
**Employment** – Employed	1.48 (1.37)	0.584	1.48 (1.36)	0.683	5.77 (1.59)	0.079	5.82 (1.54)	0.116
Unemployed	1.44 (1.42)		1.45 (1.44)		5.92 (1.61)		5.95 (1.60)	
**Relationship status** – Married/cohabitating	1.33 (1.19)	**<0.001**	1.36 (1.25)	**<0.001**	5.90 (1.58)	**0.015**	5.93 (1.56)	**0.025**
Other	1.74 (1.68)		1.67 (1.63)		5.68 (1.63)		5.74 (1.56)	
**Children aged <18 years in home** – Yes	1.35 (1.19)	**0.001**	1.39 (1.26)	**0.043**	5.96 (1.52)	**0.001**	5.98 (1.51)	**0.008**
No	1.58 (1.55)		1.54 (1.51)		5.69 (1.66)		5.76 (1.61)	

* Product referencing e-cigarettes or HTPs, respectively.

In multivariable linear regression analysis, product advertising exposure via digital media was associated with greater use intentions for e-cigarettes (β=0.24; 95% CI: 0.03–0.44) and HTPs (β=0.35; 95% CI: 0.14–0.56). Additionally, advertising exposure through POS was associated with greater HTP use intention (β=0.21; 95% CI: 0.04–0.38). Past-month use of the respective product was also associated with higher intention to use e-cigarettes (β=2.24; 95% CI: 1.87–2.61) and HTPs (β=3.24; 95% CI: 2.85–3.63), and past-month cigarette use was associated with higher intention to use e-cigarettes (β=0.59; 95% CI: 0.41–0.76) and HTPs (β=0.46; 95% CI: 0.29–0.63) (Table 3).

**Table 3:** Multivariable linear regression models examining correlates of e-cigarette and HTP use intentions and risk perceptions, cross-sectional survey of adults in Armenia and Georgia, 2022 (N=1468)

	*Use Intentions*						*Risk Perceptions*					
	*E-cigarettes*			*HTPs*			*E-cigarettes*			*HTPs*		
	*β*	*95% CI*	*p*	*β*	*95% CI*	*p*	*β*	*95% CI*	*p*	*β*	*95% CI*	*p*
Past-month product* ad exposure												
Digital media (Ref: No)	0.24	0.03–0.44	**0.023**	0.35	0.14–0.56	**0.001**	-0.07	-0.26–0.12	0.488	-0.17	-0.41–0.08	0.180
Traditional media (Ref: No)	0.26	-0.01–0.53	0.055	0.08	-0.21–0.36	0.611	-0.32	-0.55 – -0.09	**0.007**	-0.18	-0.51–0.15	0.279
Points-of-sale (Ref: No)	0.10	-0.07–0.27	0.236	0.21	0.04–0.38	**0.014**	-0.30	-0.60 – -0.004	**0.047**	-0.23	-0.42 – -0.03	**0.022**
Past-month product* use (Ref: No)	2.24	1.87–2.61	**<0.001**	3.24	2.85–3.63	**<0.001**	-0.97	-1.39 – -0.55	**<0.001**	-0.93	-1.37 – -0.49	**<0.001**
**Past-month cigarette use (Ref: No)**	0.59	0.41–0.76	**0.003**	0.46	0.29–0.63	**<0.001**	-0.86	-1.06 – -0.66	**<0.001**	-0.85	-1.05 – -0.66	**<0.001**
**Sociodemographics**												
Country – Georgia (Ref: Armenia)	-0.03	-0.27–0.20	.779	-0.03	-0.24–0.18	0.764	0.07	-0.37–0.51	0.747	0.01	-0.43–0.45	0.962
Age	-0.01	-0.01–0.00	0.058	-0.003	-0.01–0.003	0.319	0.002	0.00–0.01	0.432	0.001	-0.01–0.01	0.707
Gender – Female (Ref: Male)	-0.10	-0.26–0.07	0.235	-0.16	-0.31–0.01	0.057	0.48	0.29–0.66	**<0.001**	0.43	0.25–0.61	**<0.001**
Education – >High school (Ref: ≤High school)	-0.13	-0.28–0.02	0.098	-0.16	-0.31 – -0.01	**0.038**	0.16	-0.01–0.34	0.070	0.13	-0.04–0.30	0.139
Relationship status – Other (Ref: Married/cohabitating)	0.23	0.08–0.39	**0.003**	0.21	0.06–0.35	**0.007**	0.01	-0.17–0.18	0.948	-0.04	-0.20–0.13	0.684
Children aged <18 years in home – Yes (Ref: No)	-0.11	-0.25–0.03	0.123	-0.02	-0.16–0.11	0.735	0.20	0.04–0.36	**0.013**	0.12	-0.03–0.28	0.126

* Product referencing e-cigarettes or HTPs, respectively.

### Advertising exposure in relation to e-cigarette and HTP risk perceptions

Shown in Table 2, bivariate analyses indicated that past-month ad exposure via POS was associated with lower risk perceptions for both e-cigarettes (5.50 vs 5.92, p<0.001) and HTPs (5.38 vs 6.00, p<0.001). Other factors associated with lower perceived risk for both e-cigarettes and HTPs included lifetime and past-month use of the respective product, past-month cigarette use, and being younger, male, less educated, unmarried/not cohabitating, and without children in the home (all p<0.05).

In multivariable linear regression analysis (Table 3), past-month advertising exposure at POS was associated with lower risk perception for e-cigarettes (β= -0.30; 95% CI: -0.60 – -0.004) and HTPs (β= -0.23; 95% CI: -0.42 – -0.03). Additionally, advertising exposure via traditional media was associated with lower e-cigarette risk perceptions (β= -0.32; 95% CI: -0.55 – -0.09). Past-month use of the respective product was also associated with lower risk perceptions for e-cigarettes (β= -0.97; 95% CI: -1.39 – -0.55) and HTPs (β= -0.93; 95% CI: -1.37 – -0.49), and past-month cigarette use was associated with lower risk perceptions for e-cigarettes (β= -0.86; 95% CI: -1.06 – -0.66) and HTPs (β= -0.85; 95% CI: -1.05 – -0.66).

## DISCUSSION

This cross-sectional study explored the influence of e-cigarette and HTP marketing exposure on respective product use intention and risk perceptions among 1468 adults in Armenia and Georgia in 2022. In this sample, e-cigarette and HTP use intentions were low and risk perceptions were high; further, one-fifth reported past-month e-cigarette and HTP advertising exposure at POS, with lower exposure via digital media (about 12%) and traditional media (about 6%). Notably, advertising exposure, particularly through digital media and POS, was associated with greater intentions to use both e-cigarettes and HTPs, advertising exposure at POS was associated with lower risk perceptions for both e-cigarettes and HTPs, and advertising exposure via traditional media was associated with lower risk perceptions for e-cigarettes.

These findings suggest substantial marketing investment and inadequate regulatory enforcement. Both countries introduced comprehensive tobacco control laws (in Armenia since 2020, in Georgia since 2018) explicitly banning all types of tobacco product advertisement, promotion, and sponsorship including tobacco product display and advertisement at POS, and these bans apply to e-cigarettes and HTPs. Inadequate enforcement may be related to insufficient resources allocated to such efforts or to industry interference and marketing tactics to bypass the law, as shown in several countries^28^. Closer examination of enforcement mechanisms and potential amendments to strengthen these laws may be necessary to uphold their intended impact and close potential loopholes exploited by the industry.

Our hypothesized associations between exposure to e-cigarette and HTP advertisement and the outcomes of corresponding product use intention and risk perception were generally supported. In our study product advertising exposure via digital media was associated with greater use intentions for e-cigarettes and HTPs, consistent with the literature^6^. However, advertising exposure via digital media was not associated with perceived risk for either product in the adjusted models. This might be explained by several factors related to the nature and intended audience of digital media advertising. Unlike POS and traditional media, digital advertising platforms can provide diverse content, personalized targeting^10^, and specifically target youth by encouraging trial and emphasizing the glamour of novel tobacco products^29^ rather than health benefits.

We also found that advertising exposure at POS was associated with lower risk perceptions for both e-cigarettes and HTPs, and greater HTP use intention. POS advertising may capitalize on targeting consumers at the critical moment of potential purchase, employing visual cues and targeted messaging that highlight perceived benefits and potentially downplay associated health risks^9^. Interestingly, advertising exposure at POS was not associated with e-cigarette use intentions, which may be due to some differences in POS for HTPs versus e-cigarettes. The prominent HTP sold in these countries is IQOS, by Philip Morris International, which has developed novel marketing strategies for IQOS, including the use of special displays at POS that separate IQOS from other tobacco products, as well as chic, high-tech IQOS specialty stores.

Additionally, findings indicated that advertising exposure via traditional media was associated with lower e-cigarette risk perceptions. As suggested by the literature, traditional media channels might present selective information, possibly fostering a sense of credibility and trustworthiness, leading to reduced perceptions of risk among those exposed to these advertisements^30^. Interestingly, traditional media advertising was not associated with e-cigarette or HTP use intentions or with HTP risk perceptions, which may reflect the limited ability to deliver targeted messaging for specific groups or to identify and reach specific consumer targets via traditional media – limitations that are less relevant to the more adaptive and less expensive digital media advertising approach^31^.

Regarding the covariates, past-month cigarette use and/or respective product use was associated with higher e-cigarette and HTP use intention, likely attributable to high co-use rates^32^. Similarly, past-month cigarette use was related to lower risk perceptions for e-cigarettes and HTPs, consistent with findings from other studies in other countries^33,34^. Also, past-month use of the respective product was associated with lower risk perceptions for e-cigarettes and HTPs, which also aligns with the literature^35^; for example, one meta-analysis showed that those reporting ever using (vs never using) e-cigarettes were two times more likely to disagree that e-cigarettes are harmful and perceived e-cigarettes as less addictive^35^. These associations underscore the complexity of perceptions surrounding these products and highlights the potential impact of personal experience on individuals’ intentions and risk assessments.

Current findings have implications for research and practice. First, findings from studies of adult populations in other countries (e.g. Japan^12^, Korea^7^, US^36^) have shown similar associations between advertising exposure and risk perceptions. However, few have specifically examined the impacts of advertising via different media channels. Thus, additional comprehensive research is needed to better understand how diverse advertising channels, particularly digital media and POS strategies, are leveraged in e-cigarette and HTP marketing, and how they might differentially shape individuals’ intentions and risk perceptions regarding e-cigarettes and HTPs. Moreover, these results emphasize the urgency of enhancing enforcement of existing tobacco control laws in Armenia and Georgia to ensure the intended protection of public health. Consequently, there is a critical need for collaborative efforts between public health authorities, policymakers, and advocacy groups to expose industry marketing tactics and increase public awareness about health risks associated with e-cigarette and HTP use.

### Limitations

This sample may not represent the general adult populations of Armenia or Georgia; while the cities in this study account for about one-third of each country’s population, they do not include the two largest cities (Yerevan, Tbilisi), or more rural areas, which show different rates of smoking among men and women^22,23^. Additionally, sampling/recruitment methods across countries differed due to differences in available census data in each county, which may have contributed to the slightly different compositions by sex and smoking status. Finally, the cross-sectional nature and self-reported assessments limit the ability to make causal attributions or account for bias.

## CONCLUSIONS

This study in Armenia and Georgia indicates the influence of e-cigarette and HTP advertising exposure, primarily through digital media and POS, on increasing use intentions and reducing perceived risks of respective products among adults. Despite comprehensive tobacco control laws, significant advertising exposure persists, suggesting potential regulatory gaps. The findings emphasize the need for stricter enforcement of existing regulations and for better understanding how diverse advertising channels are used in e-cigarette and HTP marketing and their impacts. In particular, greater oversight and examination is needed for advertising via digital media, which may effectively promote use, and at the POS, which may target and influence risk perceptions. Ultimately, collaboration between civil society, regulatory entities, and government are needed to facilitate comprehensive regulation and enforcement efforts and to increase public awareness and knowledge about these products, especially among youth and young adults, and particularly as these products continue to enter and penetrate markets globally.

## Supplementary Material



## Data Availability

The data supporting this research are available from the authors on reasonable request.
