# Supplementary material for: Exposure to e-cigarette and heated tobacco product advertisements via digital, traditional media, and points-of-sale: An examination of associations with use intentions and perceived risk among adults in Armenia and Georgia
Source: Tob Prev Cessat. 2024 Oct 25;10:10.18332/tpc/191992. doi: 10.18332/tpc/191992 (PMC11504294; doi:10.18332/tpc/191992)
Supplement: Supplementary file 1 [file TPC-10-46-s1.pdf]

Supplementary Table 1. Sample characteristics

Variable	Total N=1,468 N (%) or M (SD)
Sociodemographics	
Country (N, %)	
Armenia	763 (52.0)
Georgia	705 (48.0)
Age (mean, SD)	42.92 (13.55)
Sex (N, %)	
Male	713 (48.6)
Female	755 (51.4)
Education (N, %)	
High school or less	394 (26.8)
More than high school	1074 (73.2)
Employment (N, %)	
Employed	900 (61.3)
Unemployed	568 (38.7)
Relationship status (N, %)	
Married/cohabitating	977 (66.6)
Other	491 (33.4)
Past-month cigarette use (N, %)	
No	1004 (68.4)
Yes	464 (31.6)
Children <18 in the home (N, %)	
No	744 (50.7)
Yes	724 (49.3)

©2024 Sargsyan Z. et al.
